# Involvement of microbial mats in early fossilization by decay delay and formation of impressions and replicas of vertebrates and invertebrates

**DOI:** 10.1038/srep25716

**Published:** 2016-05-10

**Authors:** Miguel Iniesto, Ángela D. Buscalioni, M. Carmen Guerrero, Karim Benzerara, David Moreira, Ana I. López-Archilla

**Affiliations:** 1 Department of Ecology, Universidad Autónoma de Madrid, 28049, Madrid, Spain; 2 Department of Biology, Universidad Autónoma de Madrid, 28049, Madrid Spain; 3 Institut de Minéralogie, de Physique des Matériaux, et de Cosmochimie, Sorbonne Universités, Centre National de la Recherche Scientifique, Unité Mixte de Recherche 7590, Université Pierre et Marie Curie Paris 06, Muséum National d’Histoire Naturelle, Institut de Recherche pour le Développement Unité Mixte de Recherche 206, 75005 Paris, France; 4 Unité d’Ecologie, Systématique et Evolution, Centre National de la Recherche Scientifique, Unité Mixte de Recherche 8079, Université Paris-Sud, Université Paris-Saclay, 91405 Orsay Cedex, France

## Abstract

Microbial mats have been hypothesized to improve the persistence and the preservation of organic remains during fossilization processes. We test this hypothesis with long-term experiments (up to 5.5 years) using invertebrate and vertebrate corpses. Once placed on mats, the microbial community coats the corpses and forms a three-dimensional sarcophagus composed of microbial cells and exopolymeric substances (EPS). This coverage provides a template for i) moulding superficial features, resulting in negative impressions, and ii) generating replicas. The impressions of fly setulae, fish scales and frog skin verrucae are shaped mainly by small cells in an EPS matrix. Microbes also replicate delicate structures such as the three successive layers that compose a fish eye. The sarcophagus protects the body integrity, allowing the persistence of inner organs such as the ovaries and digestive apparatus in flies, the swim bladder and muscles in fish, and the bone marrow in frog legs. This study brings strong experimental evidence to the idea that mats favour metazoan fossilization by moulding, replicating and delaying decay. Rapid burial has classically been invoked as a mechanism to explain exceptional preservation. However, mats may play a similar role during early fossilization as they can preserve complex features for a long time.

A wealth of taphonomic studies has shown that processes enabling fossilization are uncommon, especially for soft tissues^1^,^2^. Based on the frequent association between microbial biofilms, articulated fossils and mineralized soft tissues in many deposits like Konservat Lagerstätten, it has been suggested that microorganisms are an important factor for exceptional preservation^3^,^4^. This suggestion is highly supported by thorough observation and analysis of the fossil record^4^,^5^,^6^,^7^,^8^,^9^,^10^,^11^,^12^. Moreover, several taphonomic studies have shown that the presence of microbes can positively affect preservation during early diagenesis^13^,^14^. The experimental contributions made in this field have focused on characterizing the processes that microorganisms stimulate in the preservation of organic remains (*i.e.*, capture, protection, inhibition of decay, mineralization and pseudomorphing (as summarized by Briggs, 2003a)), and the microenvironmental conditions necessary for these processes to occur^13^. Most of these experimental approaches conducted so far have used heterotrophic bacteria and/or biofilms inoculated in different media (*e.g.*^13^,^14^,^15^). By contrast, only few taphonomical experiments have used natural microbial mats. In this context, it is important to notice that biofilms present remarkable differences with microbial mats. In particular, biofilms have a simpler biological composition with a relatively small number of microbial species, whereas mats are complex communities, frequently comprising hundreds of different species including photosynthetic oxygenic cyanobacteria, red and green anoxygenic photosynthetic sulphur bacteria, and a large variety of heterotrophic bacteria. Thus, mats contain multiple trophic levels (*i.e.*, primary producers, consumers, and decomposers) and can be considered as genuine ecosystems^16^. As a result, mats develop a variety of metabolic reactions, some of them producing steep physico-chemical gradients which are not as well developed in biofilms. Therefore, the outcomes of taphonomic experiments may likely be very different depending on whether biofilms or mats are used. For example, Darroch *et al.*^17^ tested the possible implication of microbial mats in the genesis of death masks favouring the preservation of Ediacaran fossils. Moreover, Iniesto *et al.*^18^ and Guerrero *et al.*^19^ observed the formation of a microbial mat sarcophagus that covered carcasses, in parallel with a remarkable slow-down in the decay and the development of different microenvironments around carcasses^20^ supporting that microbial mats may have a significant impact on early fossilization. Nevertheless, the comprehension of the mechanisms and processes leading to exceptional preservation requires more experimentation.

Moulds and replicas are widespread fossilization products. Gehling^21^ postulated a fossilization pathway with an important role of microbial mats in the preservation of impressions by the formation of impressions that maintained the shape of the specimens. By the formation of a mould, a negative copy of the external body parts (*i.e.*, an impression) is generated. The implication of microorganisms in mould formation has been suggested by numerous observations of fossils: microbial mats might be involved for instance in the preservation of dinosaur skin^22^,^23^, of body-shape, integument tissues and colour patterns^24^, in the formation of the skin shadow in ichthyosaur fossils^25^, and even in the preservation of ancient human footprints^26^. Mould formation was most likely crucial also for the conservation of soft-bodied organisms^21^,^27^ through the mat-mediated cementation of sediment around bodies, which allowed preserving their external morphology by authigenic mineralization^17^,^28^,^29^. In addition, replication results from intensive microbial colonization that replaces and reproduces the original tissues, followed by stabilization of the fossil within the sediment. Preservation through replication by microbes has rarely been described and is limited to few examples, such as the endoreplica produced during the coating process, which replicates the pliable vascular structures and osteocytes of bones^30^. Another remarkable example comes from experimental embryo fossilization by thin heterotrophic biofilms, which replace and replicate the morphology of the consumed embryo^14^.

In contrast with simple biofilms, microbial mats are organized in layers, each one with specific community composition and metabolic functions^31^,^32^,^33^. The upper layers, typically dominated by photosynthetic species, can be associated with the delay in decay thanks to the formation of a dense sarcophagus, which generates an special physico-chemical environment and also protects against abiotic factors such as water flow^4^,^10^. Furthermore, mineral precipitation may occur in all layers. For example, Schiffbauer *et al.*^34^ showed that bacterial sulphate reduction, a metabolism that is present typically in the anoxic deep layers, can induce the pyritization of fossils, while Iniesto *et al.*^35^ evidenced the formation of Mg-rich silicates within the green upper layer of an experimental mat fossilizing fish. The present study aims at understanding the involvement of microbial mats in animal body preservation through the monitoring of changes that occur during the decay of carcasses placed in tanks with microbial mats to observe the formation of imprints and replicas. The tested taphonomic phase extends from the subaerial exposure of a body to its complete coverage by the microbial mat, prior to burial within the sediment. These long-term experiments, up to 5.5 years, were carried out using flies (*Musca domestica*), fish (*Carassius auratus* and *Paracheirodon innesi*) and frogs (*Hymenochirus boettgeri*). We imaged inner soft-tissues to assess the persistence and the potential invasion of the microbial mat within the animal bodies. We specifically focused on the phototrophic oxic layer (top level of the mat) to show that its intrinsic properties and functions are critical in the tapho-generation of moulds and replicas within a microbial sarcophagus.

## Results

### Formation of sarcophagi

**Figure 1:**
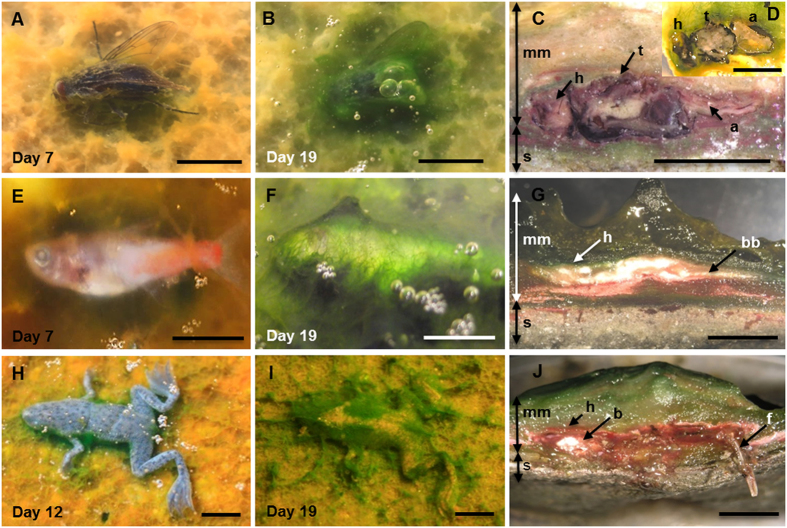
Temporal sequence of the microbial coating of carcasses. (**A**–**D**) Fly (*Musca domestica*); (**E–G**) Fish (*Paracheirodon innesi*), (**H–J**) Frog (*Hymenochirus boettgeri*). The left column shows the organisms after one week on the mat. The central column corresponds to carcasses completely covered at day 19. The right column shows the sections cut across microbial mat blocks containing the carcasses. Note the coherence of the sarcophagus around the bodies of flies (**C**, 5.5 years and **D**, 8 months), fish (**G**, 8 months) and frog (**J**, 12 months). Abbreviations: a, abdomen; b, brain; bb, back bones; h, head; f, femur; mm, microbial mat; s, sediment; t, thorax. (Scale bar: **A–D**, 5 mm; **E–J**, 10 mm).

**Figure 2:**
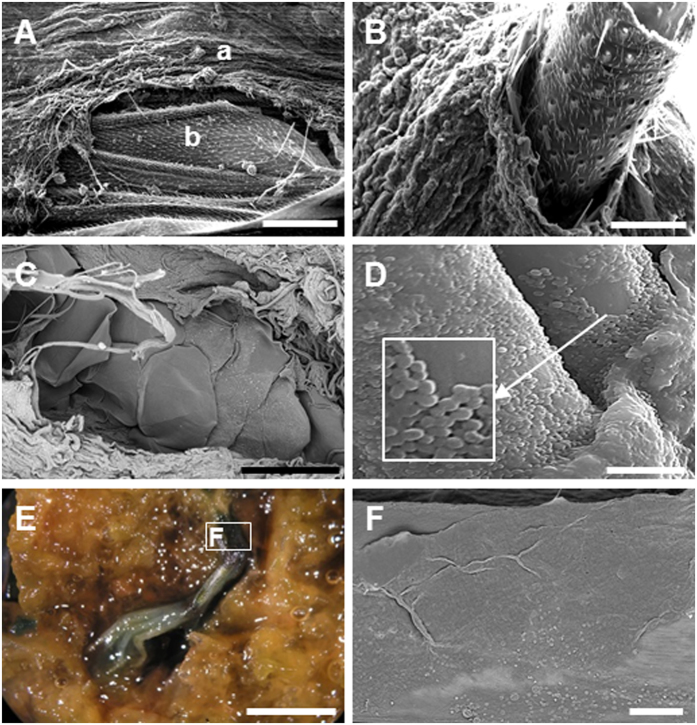
Microbial mould of the external shape of carcasses. (**A**) SEM image of the fly wing (8 months): a, microbial coverage; b, wing. (**B**) Microbial sheath coating the fly femur. (**C**) Neon tetra fish tail, covered by a thick mat layer after 8 months. Fish scales are visible in the centre of the image. (**D**) Red carp fishbone after 15 months; the EPS matrix and coccoid bacteria are distinguishable. (**E**) Image under the binocular of frog foot and toes trapped within a mat, after 8 months. (**F**) SEM detail of the leg, showing the preserved surface of the skin. (**A–D**,**F**) are SEM images. (Scale bar: **A**, 200 μm; **B**, 50 μm; **C**, 500 μm; **D**, 10 μm; **E**, 5 mm; **F**, 150 μm).

Fly, fish and frog bodies were placed on the surface of experimental microbial mats grown in tanks, as well as on the surface of sediment for control experiments (for more details, see section 4). The mat microbes completely covered the animals after one to three weeks depending on the type and size of the animal (the smaller the size, the faster the coverage; see Supplementary Fig. S1 for a detailed summary of the sequence of coverage and preservation in mats). Despite these time variations, the process always followed the same pattern. After carcasses were laid on mats, cyanobacterial filaments from the upper layer of the microbial community started to grow on the bodies and trapped them (Fig. 1). After this stage, an increasingly thick microbial veil grew over the animals and formed the final three-dimensional sarcophagus (Fig. 1C,D,G,J). Scanning electron microscopy (SEM) observations allowed the detailed description of this coating process and revealed the occurrence of abundant exopolymeric substances (EPS) produced by the microbial mats (Fig. 2). The dense and consistent EPS-rich matrix was a major constitutive element of the developing sarcophagus and embedded different microbial cells observed in the course of the experiment. In the case of flies, the microbial veil developed as a sheath over the carcasses, including wings (Fig. 2A) and legs (Fig. 2B). A consistent coat was also observed over fish bodies (Fig. 2C shows a detail around the fish tail region). The removal of that microbial coat allowed to observe that, after 15 months, fish spines were also coated by a film of coccoid-shaped bacteria (Fig. 2D). Similarly, the complete articulation of frog legs could be observed once the top layer was removed (Fig. 2E). On the frog skin, which remained remarkably conserved even after 12 months (Fig. 2F), the presence of few coccoid and filamentous bacterial cells was detected.

After the full coverage of the bodies by the mat microorganisms was achieved, the microbial layer continued to grow until becoming a dense sarcophagus that reproduced the three-dimensional shape of the animal bodies. This process was accompanied by changes in body dimensions that varied for the different species examined. The size of the head and thorax of flies remained constant within the sarcophagus, whereas the softer abdomen flattened over time (Fig. 1C,D). Fish bodies, covered by scales, were also quite resistant against flattening (Fig. 1G) but frogs showed an obvious reduction of body thickness (Fig. 1J).

### Formation of imprints and replicas

**Figure 3:**
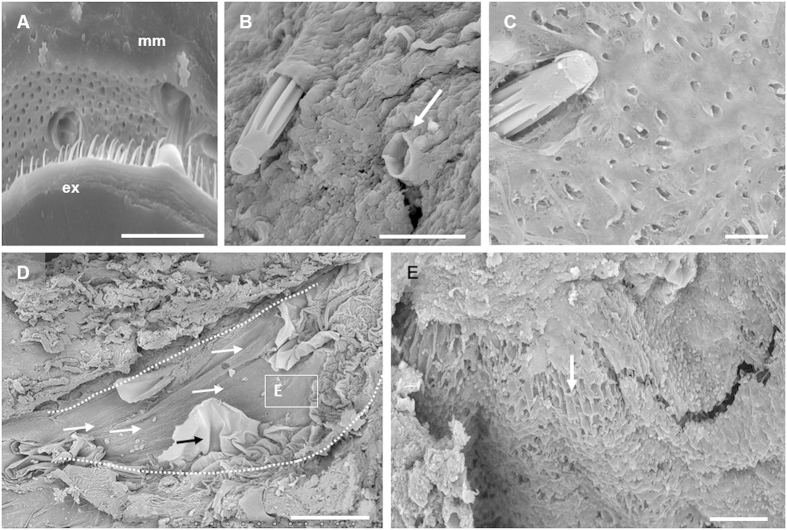
SEM images of the finely detailed imprints on the mat. (**A**) Detail of the fly exocuticle setulae (ex) and their imprints on the microbial mat (mm). (**B**) Fly s*etae* penetrating the microbial coverage; note the preservation of imprints even in the case of lost *setae* (arrow). (**C**) *Setae* trapped in a microbial mat and impressions of *setulae*. (**D**) Impression of the fly wing; white arrows point to the vein impressions. The black arrow indicates the original wing tissue trapped in a microbial layer and the dotted white line shows the outline of the wing. (**E**) Pattern of holes left by the wing hairs as an impression on the mat (arrow). (**A**,**D**,**E**) 8 months fly; (**B**,**C**) 5.5 years fly. (Scale bar: **A–C**,**E**, 20 μm; **D**, 500 μm).

Microbial mats formed a negative print of the surface morphology of bodies, namely an impression, with high fidelity even at very fine scale. Fly exocuticles, which contain many hairs of different sizes and morphologies, provide a particularly outstanding example (Fig. 3A–C). Microbial sheaths that surrounded the setae (biggest hairs) remained intact after the removal of the corpse (Fig. 3B). In addition several regions of the mat presented a multitude of small impressions and holes corresponding to the setulae (hairs smaller than setae) (Fig. 3C). Even very delicate structures, such as wings, left an impression preserved by the mat. Not only the wing shape, but also the relief of the venation pattern (Fig. 3D) and the wing hairiness (Fig. 3E) were copied on the mat surface.

**Figure 4:**
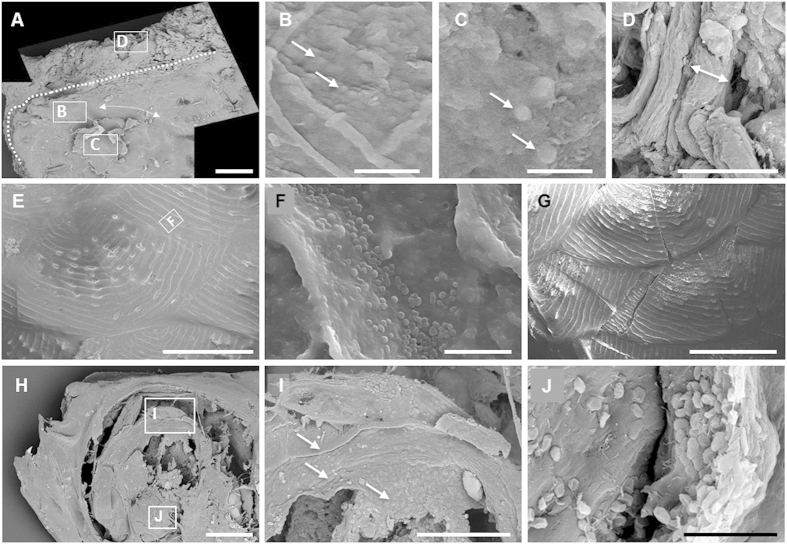
SEM images of the impression of fish bodies on the microbial mat during the entombment process. (**A**) Mould of the head of a neon tetra fish after 24 months; three spots are marked and magnified in the following panels (**B**–**D**). Dotted line: imprint of the head; arrows: circular edge of the eye. (**B**) Impression of the eye with filamentous and rod-shaped cells (arrows) embedded within the EPS matrix. (**C**) Detail of the replica of the eye lens which was trapped in the mould after the removal of the head. Arrow points small coccoid cells (**D**) Regular microbial mat in an area outside the imprint, composed mainly of packages of *Coleofasciculus* filaments (see the package indicated by arrows). (**E**) Impression of the scales of a red carp after 15 months. (**F**) Detail of the impression of a fold in **E**. (**G**) Fish scales with an exceptional conservation of the original organization. (**H**) Head of neon tetra fish (24 months) where the eye structure seems to be still preserved (see panels **I**,**J**). (**I**) Detail of the superposition of microbial layers that replicate and replace the original eye tissues. Arrows: layers composed of an EPS matrix with embedded bacteria. (**J**) Replica of the eye lens replaced by microbes and EPS matrix. (Scale bar: **A**,**E**,**G**,**H**, 500 μm; **B**,**C**,**F**,**J**, 10 μm; **D**, 50 μm; **I**, 200 μm).

Fish bodies were also printed on the mats. The head impression clearly highlighted the eye and lens surfaces (Fig. 4A). In both cases, the printing showed fine microbial filamentous and slightly bacillary cells embedded in an EPS matrix (arrow, Fig. 4B,C, respectively). By contrast, the top of the mat that was not in direct contact with the fish body showed a messy package of large filaments of the cyanobacterium *Coleofasciculus* (Fig. 4D). Scales were also printed on the mat with fine detail, and a succession of overlapping and organized semicircles was clearly visible after carcass removal (Fig. 4E). Coccoid bacteria could also be distinguished at higher magnification (Fig. 4F). This imprint corresponded to the external relief of the original fish scales, characterized by concentric lines (Fig. 4G). Energy-dispersive X-ray spectrometry (EDXS) confirmed the calcium phosphate composition of the scales, in contrast with the organic composition of the impression (see Supplementary Fig. S2).

Strikingly, fish eyes remained clearly recognizable even after 24 months within the mat (Fig. 4H) and showed the lens and three layers (Fig. 4I) likely corresponding to the conjunctiva, cornea and iris. The observation of these areas at higher magnification revealed that each layer was actually a replica formed by numerous short rod-shaped bacterial cells embedded in EPS (Fig. 4J).

**Figure 5:**
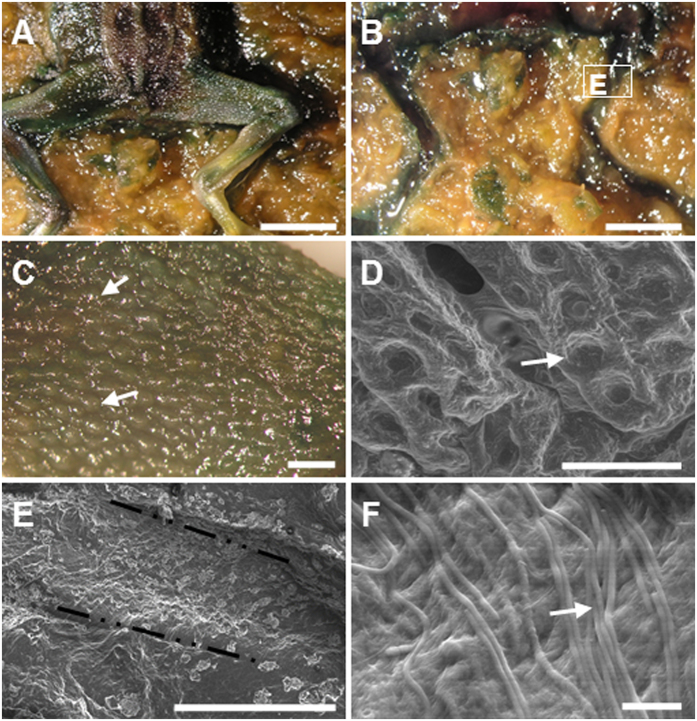
Formation of the impression of frogs on the microbial mat. (**A**) Frog laying on the mat after 12 months once the upper layer of the sarcophagus was removed. (**B**) Hind limb impression. (**C**) Detail of the skin showing verrucae (arrows) after 8 months. (**D**) Environmental SEM image of the verrucae impressions (arrow) on the microbial mat. (**E**) SEM image of the microbial mould of a leg after 12 months. (**F**) Microbial filaments at the base of the imprint. (Scale bar: **A**,**B**, 2 mm; **C–E**, 500 μm; **F**, 20 μm).

Frogs covered by mats underwent a substantial reduction of body thickness after 12 months but the tegument remained intact, enabling the formation of an accurate impression of the whole carcass on the mat surface (Fig. 5A,B). The soft skin of frogs leaved a clear imprint in the mat and typical structures of the skin surface, such as the conspicuous aligned verrucae (Fig. 5C), were also visible as negative prints on the mat (Fig. 5D). SEM observation of the mat around the legs (Fig. 5E) showed visible cyanobacterial-like filaments embedded in an EPS matrix (Fig. 5F).

**Figure 6:**
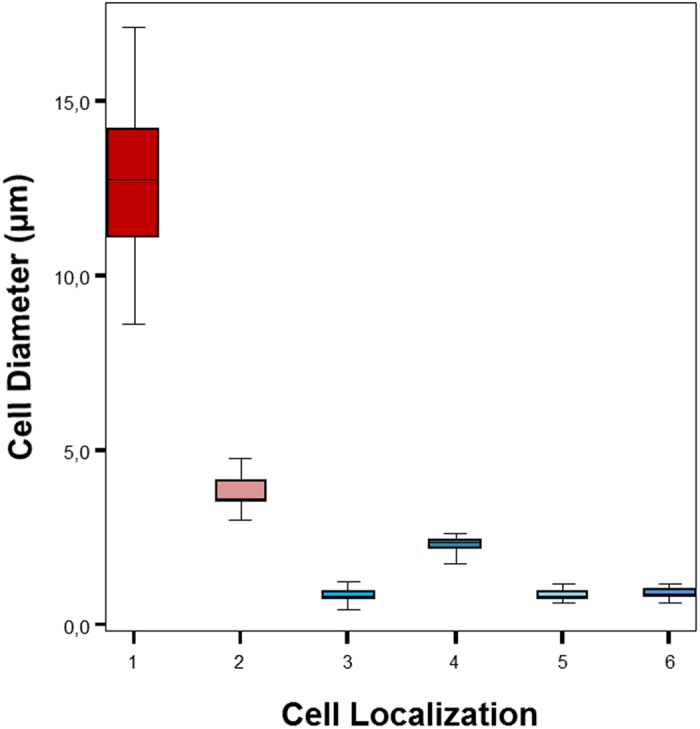
Box-plot built with the measurements of cells present in SEM images of body impressions of fly wing (N = 118), fish scales (N = 270) and bones (N = 130), and frog skin (N = 74) and from the upper layers of the mat far from the animal bodies (N = 90). Localization: 1, *Coleofasciculus* thick packages (see Fig. 4D); 2, other cells in the upper layers of the mat; 3, cells over fish scales (see Fig. 4F); 4, filaments in the mould of the frog (Fig. 5F); 5, cells over fish bones (see Fig. 2D); 6, cells in fly impression (Fig. 3E). Upper and lower fences represent Q3 + 1.5 IQR (InterQuartil Range) and Q1 - 1.5 IQR respectively. Median is represented into the box by a black line.

A careful observation of impressions evidenced different sizes of microbial cells depending on their distance to the bodies. We measured the diameter of cells on the body impressions of the fly wings (N = 118), the fish scales (N = 270) and bones (N = 130), frog skin (N = 74), as well as cells from the upper layers of the mat not in direct contact with the bodies (N =  90). At the upper layer of the mat, the *Coleofasciculus* packages measured 12.77 ± 2.11 μm in thickness, whereas other smaller cells present in this layer had a diameter of 3.65 ± 0.79 μm. The size of cells composing the mould of the fly wing, scales and over fish bones ranged between 0.8 and 1 μm (Supplementary Table S1), although one smaller cell group (around 0.7 μm) was observed in the case of scales. Filaments present in the mould of the frog measured 2.27 ± 0.21 μm in thickness, being also smaller than cells lying on the top (Supplementary Table S1). Figure 6 shows a box plot that summarizes the different cell size populations according with the different localizations.

### Conservation of carcasses and inner soft parts

**Figure 7:**
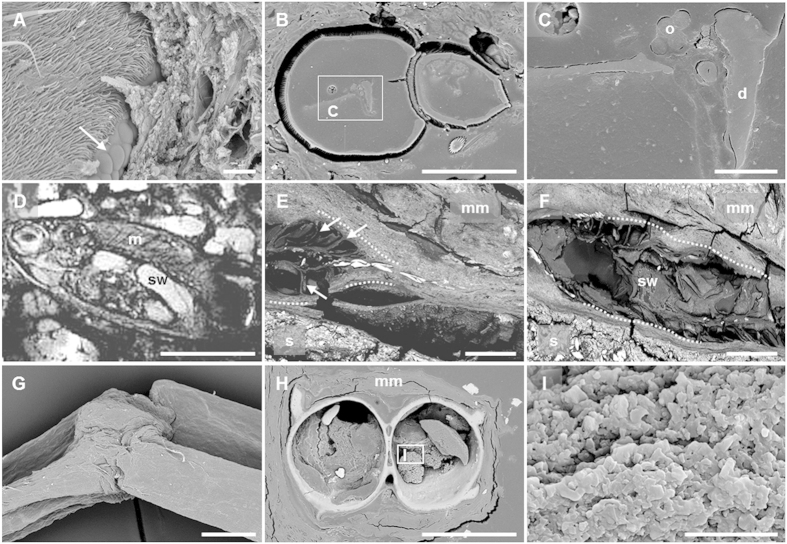
Preservation of animal tissues after prolonged periods of residence in the microbial mats. (**A**) Ommatidia (arrow) of a fly after 5.5 years. (**B**) Epoxy-embedded section of a fly (abdomen and thorax) after 5.5 years. Light grey structures inside it correspond to preserved inner soft tissues. (**C**) Detail of soft tissues preserved in the abdomen. According to the size, organization and position, these organs are most likely the ovary and digestive tract. (**D**) MRI of an exceptionally preserved fish (*Paracheirodon innesi*) after 8 months. (**E**) Tail of a 24-months preserved fish. Arrows: supporting tissues around the vertebral column; (**F**) Section of a fish completely embedded in the microbial mat (24 months). Dotted lines in (**E**,**F**) indicate the external shape of the fish. (**G**) Fully articulated leg of a frog after 12 months. (**H**) Epoxy-embedded section of the tibia and fibula. (**I**) Detail of the preserved bone marrow in (**H**) where microorganisms appear to be absent. Images acquired by SEM (except **D**). Abbreviations: d, digestive tract; m, muscle boundless; mm, microbial mat; o, ovary; s, sediment; sw, swim bladder (Scale bar: **A**,**C**, 20 μm; **B**, 100 μm; **D**, 5 mm; **E–H**, 500 μm; **I**, 10 μm).

Animal bodies were degraded gradually within the microbial mats but the decay rate was much slower than that observed for corpses maintained in control tanks containing sediment without microbial mats, in agreement with previous experiments using fish bodies^18^. Flies, which have a hard cuticle, retained their exoskeleton practically intact even after 5.5 years within the mat. Even complex organs, such as the fly ommatidia, still conserved their original organization after this long time (Fig. 7A), and some inner soft-tissues were still present (Fig. 7B). In fact, although internal structures were in general difficult to identify, some of them, most likely the ovary and the digestive tract of flies, were visible in resin-embedded sections (Fig. 7C). By contrast, flies in control experiments without microbial mats were completely degraded after 6 months. Inner tissues were also observed in the fish bodies covered by mats. After 8 months, magnetic resonance imaging (MRI) revealed the presence of the swim bladder and muscle bundles (Fig. 7D) and, after 24 months, fish bodies kept the articulated backbone (Fig. 7E) and the swim bladder (Fig. 7F), as observed by SEM in resin-embedded sections. In frogs, the articulation of the skeleton remained unbroken (Fig. 7G) and the skin maintained its integrity. Delicate structures, such as the interdigital membrane (Supplementary Fig. S3A,B) and the small protuberances on the skin surface (Supplementary Fig. S3C,D), persisted after 12 months. The section of the frog femur showed organic tissue inside, likely rests of the bone marrow that persisted after 12 months (Fig. 7H,I). No microorganisms appeared to be present in this section. Prokaryotic cells seemed to be also generally absent inside fish and flies.

## Discussion

Our experiments show that the formation by the microbial mat community of a sarcophagus composed of microbial cells embedded in an EPS matrix around carcasses has two important consequences regarding the initial phases of the taphonomic process. First, the presence of microbial mats reduces the time of the entombment of carcasses to a few days (less than 20 days in our experiments) and generates a physical and chemical environment distinct from ambient conditions. Second, the sarcophagus provides the template for the formation of moulds and imprints, and it promotes the replica of soft organic structures.

In addition, our observations illustrate three important facts: the coherence of the mat upper layer, necessary for the imprint formation once the body has been covered; the presence and distribution of small coccoid cells around the carcasses; and, finally, the conservation of inner soft tissues even after long periods of residence of the bodies within the mats. As explained above, the coat formed on animal bodies by microbial cells embedded in the EPS matrix is able to copy the shape and details of positive structures such as the fly wing venation, the fish scales or the verrucae of the frog skin. Moreover, very fine details such as the 3–4 μm impressions left by the fly setulae and wing hairiness can also generate imprints. The implication of microbial mats in the formation of high-fidelity impressions is widely accepted (*e.g.*^21^,^27^,^36^), and Darroch *et al.*^17^ have highlighted experimentally the formation of “death masks” by mats, developing the model proposed by Gehling^21^. This model assumes a differentiated preservation of the diverse parts of the carcasses since the impression of the bottom surface, which is in direct contact with the mat, is favoured. However, in our experiments, imprints were observed on both the top and the bottom of the carcasses.

Small coccoid or slightly elongated prokaryotic cells less than 1 μm in size (usually embedded within the dense EPS matrix) appeared either coating structures (such as the fish bones) or forming part of the impressions. Small filamentous prokaryotic cells, with an average diameter of 2 μm, were also present in moulds of frogs. The size of prokaryotic cells seemed to be different depending on the proximity to the carcasses: large cells (such as *Coleofasciculus* thick packages) were located distant from the bodies, while small cells were in close contact with them. Further studies will be needed to clarify the functional role of the different prokaryotic species along the microbial succession during early diagenetic processes. The occurrence of small spherical structures in the fossil record has been frequently interpreted as coccoid prokaryotic cells. This is the case of the dinosaur *Pelecanimimus* from the Early Cretaceous Konservat-Lagerstätten of Las Hoyas (Spain), which was covered by a purported phosphatized microbial mat containing layers of coccoid cells embedded in a film^22^. It was shown that the microbial mat replicated the structure of the muscles and skin of this dinosaur. In addition, negative impressions of coccoid and rod shaped bacteria were observed on the bone surface of teleostean fish from the same deposit^37^. In the Miocene locality of Libros (Spain), articulated skeletons of frog and salamander specimens were discovered enclosed in a thin carbonaceous bacterial biofilm with fossilized coccoid-shaped or slightly elongated bacterial cells^38^. The horseshoe crab fossils from the Upper Jurassic Nuspligen site (Germany) also showed mineralized biofilms with coccoid and spiral forms in association with muscles^39^. In the present study, we observed bacteria with the same shape and size in the replicas of fish eyes, in agreement with what has been observed in fossils. In fact, Martill^25^ and Gupta *et al.*^37^ described carbonaceous laminae formed by a microbial film replacing vertebrate eye soft tissues in marine and alkaline Mesozoic waters. Moreover, melanosomes, which are organelles present in most animal eyes, have been recognized in a Lower Cretaceous bird^40^ and in an Early Eocene fish^41^. However, the distinction between melanosomes and microbes is controversial^42^. Taking into account the distribution and density criteria proposed by Moyer *et al.*^42^ to differentiate them, we consider that the structures observed in the present work were most likely prokaryotic cells.

The microbial mat sarcophagus protects the animal bodies and outlines their detailed three-dimensional shape and it may also be involved in subsequent (bio)mineralization. In fact, EPS compounds, which are a major component of the sarcophagus, have been shown to provide nucleation centers for mineral precipitation^33^,^43^. Even the interior of animal bodies is protected from substantial decay. This is evidenced by the persistence of the ovary and digestive tract in flies or the swim bladder in fish after long periods (up to 5.5 years in the case of flies). The persistence of digestive tract tissues mediated by microbes has already been highlighted experimentally by Butler *et al.*^44^ but it had never been evidenced before in the case of sexual organs. Previous experiments conducted by Briggs and Kear^45^,^46^ using heterotrophic microbes explained this delayed decay by the development of anoxic conditions around the bodies after only two months. In the case of mats, much more complex microbial communities, the implication of anoxia in tissue persistence could be important in the earlier phases of coverage (several days). However, internal fish tissues, which persisted over 1000 days, become oxic after three months^20^. This oxic phase is important since in control carcasses over sediment, in which anoxia persists, complete decomposition occurs (see Supplementary Fig. S1). Moreover, frog bones still have an intact medulla after 12 months. This kind of tissue is seldom preserved and, therefore, very rare in fossils. Noticeable exceptions are the observation of medulla in dinosaurs, such as *Triceratops horridus*^47^ or *Tyrannosaurus rex*^48^, and in the anuran *Pelophylax pueyoi*^38^ (formerly named *Rana pueyoi*), this last one likely related with mat-mediated preservation.

As mentioned above, it has been suggested that the sealing effect of the microbial mat can be even more important because the whole EPS organic matrix may work as a chelator of cations and then drive mineral crystal nucleation^49^,^50^,^51^. In fact, the relevant role of the mat matrix in preservation has been highlighted by Schiffbauer^34^. In a previous work, we described a Ca-rich thin veil on fish specimens after only 1 month of incubation within the mats^18^. Hence, the microbial activity may generate specific microenvironments favouring mineral precipitation and further protection against degradation (*e.g.*,^52^). Although the occurrence of biomineralization in taphonomic experiments has been linked frequently to acid conditions^45^,^53^, this assumption may not be correct in the case of microbial mats. In fact, preservation can be initiated through other phases, such as carbonates^54^, calcium phosphate^55^ or Mg-rich silicates^56^, which precipitate preferentially in basic and oxic conditions, as those already described during carcass preservation in mats^20^. For example, we have described the occurrence of a talc-like mineral phase in intimate contact with the upper face of a fish embedded in a mat for 5 years^35^. Precipitation of those mineral phases at the interface between carcasses and mats would likely enhance the long-term preservation of impressions, thus increasing the probability of fossilization. According to our observations, the fossil preservation of impressions in mats would follow three main steps: 1) the organic remain is embedded by the mat microbial community, 2) the surface of the body generates a detailed impression on the mat, and 3) the precipitation of a mineral phase stabilizes the impression. The body as well as its mould may subsequently be buried by sediments. At this point, posterior fossilization stages would depend primarily on abiotic factors such as the mineral phase generated, later metamorphism, etc.

Our results support that microbial mats can be relevant for two major taphonomic processes: replication and moulding. Their occurrence depends on the ecological factors affecting mat growth. It is noteworthy that, according to their palaeoecological description^10^,^57^, several relevant Konservat Lagerstätten (*e.g.* Libros or Las Hoyas) presented environmental conditions that were advantageous for the development of microbial mats (*i.e*., presence of shallow lakes and ponds, optimal water conditions and seasonality, high pH, etc.) at the time when their fossils were formed. Fossils from several Konservat Lagerstätten show features similar to those that we observe in our experiments. For instance, the persistence of insect carcasses herein described can explain the preservation of arthropod fossils such as those found in Kishenehn (Eocene, USA)^58^ and Crato Formation (Early Cretaceous, Brasil)^59^. The exceptional fossilization of ommatidia has been reported in trilobites from sites of different ages, ranging from Cambrian to Carboniferous^60^. Moreover, the cast of external features of *Pelecanimimus*^22^ is an unequivocal example of the moulding ability of mats described in our work. This also applies to the carbonate-based anuran outlines studied by McNamara *et al.*^54^,^57^. Actually, the formation of imprints on both sides of the carcasses and the preservation of external morphologies are the result of the inherent features of the microbial sarcophagus (*i.e.*, coherence, full coverage of the body, microenvironmental conditions and high-fidelity impressions). Our work identifies processes of preservation that may explain many characteristics of the fossil record in sites with indications of contemporary microbial mat growth. For example, several Ediacaran fossils may be interpreted as additional examples of the implication of microbial mats in replication and moulding^61^. In fact, based on our results, we consider that a microbial sarcophagus might be formed not only over dead organisms but probably also over sessile ones. McIlroy *et al.*^62^ have shown with sessile organisms that they are able to generate superficial impressions in the surface of photosynthetic mats. In addition, “flinders-style preservation” contemplates the formation of positive and negative impressions^27^ which is consistent with our results. The microbial sarcophagus would help to enhance the formation of impressions, and would increase the resistance of bodies to collapse. According to experimental evidence and the study of the fossil record, the assumption that microbial mats are crucial for these pathways of microbially-mediated preservation is plausible.

Quick burial and delayed decay are two conditions considered to be compulsory for exceptional preservation^63^. Traditionally, rapid burial was most often supposed to occur by sudden entrapment within sediments. However, microbial mats offer an interesting alternative as these microbial communities can rapidly cover carcasses, and eventually also living sessile organisms, by the colonization of the body surface, leading to the formation of a sarcophagus. This microbial coverage protects the body during the subsequent burial stages. In addition, mats also fulfil the second condition as they dramatically slow down the decay of the bodies. The formation of a sarcophagus protects and preserves carcasses inside mats for very long periods, allowing the possibility of a subsequent burial in sediments not necessarily very rapid. Our experiments show a rapid growth of mats around the bodies and a notable delay of their decomposition for three types of organisms, both vertebrates and invertebrates. This suggests that persistence and preservation in mats can be a general phenomenon. Moreover, microbes appear to be involved in the replication of original structures. The direct contact of the prokaryotic cells (and their secreted EPS) with carcasses creates accurate impressions of their surfaces. This intimate relationship between a macroscopic organism and microbial mats is most likely capital for its preservation (Supplementary Fig. S4). Despite the progress in experimental taphonomy, mineralization of replicas and impressions still remains poorly understood. Longer experiments will be needed to further characterize the relationships existing between the metabolic activity of the microbial community and early diagenesis.

## Material and Methods

Original mat samples were taken at Lake Salada de Chiprana (Zaragoza, Spain), which is a hypersaline (30–70%) permanent shallow lake of endorheic origin in a semi-arid region of the Ebro depression (Aragon, NE Spain)^64^. The lake is fed by ground water discharge, which provides a source of abundant magnesium sulphate (up to 700 meq.L^−1^ of SO_2_^4−^ and Mg^2+^) and sodium chloride (approximately 300 meq.L^−1^). The mats from the Salada de Chiprana are dominated by the filamentous cyanobacterium *Coleofasciculus chthonoplastes* (Thuret ex Gomont) M. Siegesmund, J.R. Johansen & T. Friedl^65^ (formerly known as *Microcoleus chthonoplastes* Thuret ex Gomont), which forms the framework of the mats. The upper layer of the mat is mainly formed by the taxa *Chloroflexus*, *Coleofasciculus* and *Pseudoanabaena*-like, while the red layer below is composed of anoxygenic photosynthetic bacteria, and the dark layer in the bottom is made up of anaerobic microbial populations and dead cyanobacteria^66^. Chiprana mats were prepared as described in previous experiments^18^ and placed in three 50 × 25 × 20 cm glass tanks containing a 2–3 cm base of limestone overlain by a 3–4 cm layer of sediment from the lake, and grown in the laboratory. Tanks were also filled with water from the lake in order to get a 2 cm water column (details of the ionic water composition can be checked at^35^). Chambers were illuminated with a constant light output and a cold beam (OSRAM Decostar 51 Titan) with a photoperiod similar to the natural one (adjusted to 10 h of daylight). Three additional tanks, used as controls, were prepared following the same procedure but without the microbial mat overlaying the sediment. Control tanks were kept in dark in order to avoid the development of a microbial mat from the resting stages of photosynthetic populations present in the sediment. All the tanks were placed in a controlled-temperature room. During the experiment, temperature in tanks was 23 ± 0.5 °C, and pH was 8.6 ± 0.7 in tanks with mats and 8.3 ± 0.5 in non-mat tanks. Conductivity (50.1 ± 2.3 mS·cm^−1 ^and 52.2 ± 1.8 mS·cm^−1^ for mat and non-mat tanks, respectively) and water depth (about 2 cm) were kept almost constant in tanks by the periodical addition of sterilized distilled water to compensate for water evaporation.

Three groups of animals were tested in order to identify possible differences in mould formation: one arthropod (fly) and two vertebrates (fish and anuran). The arthropod was *Musca domestica* (grown in the laboratory from wild specimens). Fish species were the red carp (*Carassius auratus*) and the neon tetra fish (*Paracheirodon innesi*) and the anuran was the “nailed small toad” (*Hymenochirus boettgeri*). All animals were euthanized with tricaine mesylate (MS-222, 0.06%) diluted in TRIS (0.29%) following the Animal Care Protocol of the Universidad Autónoma de Madrid (U.A.M.). All the experimental protocol was checked and approved by the authorized body of the government of the Comunidad de Madrid (Clinical Research Ethics Committee of the U.A.M.: http://www.uam.es/otros/ceiuam/), which evaluates protocols and projects that include experiments with animal. Carcasses were laid on the surface of microbial mats. Flies, fish and frogs were placed in separated tanks. Control carcasses were placed in independent tanks without mat. The microbial covering process was monitored over the whole experiment time. According to previous experiments, bodies were distributed on the mat surface with a lateral distance of 4 cm in order to avoid interferences between them. To examine the progress of mould formation, arthropod samples were examined at two times: 8 and 66 months (5.5 years). The red carps were observed at month 15, neon tetra fish at months 8 and 24, and anurans at months 8 and 12. Two specimens were studied for each species at each time. To observe these specimens already embedded inside the microbial community, blocks of the mats containing the animal bodies were extracted from the tanks. When the mats were easily detachable from the bodies, the microbial cover was removed to observe the carcass surface and the microbial veil in direct contact with it. Blocks were also cut at the centre to get two parts where the carcass was exposed. One of the parts was emptied by removing the carcass in order to analyse the sarcophagus generated by the mat and the imprint formation. The other part allowed the observation of the relation between the mat coverage and the carcass as well as the inner tissue persistence. In every case, a first observation of carcasses and moulds with a binocular magnifier was carried out. More detailed observations were achieved using Scanning Electron Microscopy (SEM). Because of the inherent features of the mat, in particular its dense EPS layer, chemicals used in sample preparation have difficulties to penetrate inside the block; this is why, after previous trials carried out in our laboratory, a specific protocol for microbial mats was established. Following these trials, fly, fish, and anuran samples were fixed in vacuum at room temperature during 64 h with 2% glutaraldehyde and dehydrated in ethanol at increasing concentrations (30%, 50%, 70%, 90%, 3 × 100%, 60 min each step) and dried overnight at 37 °C. To obtain precise details, several samples were embedded in epoxy resin and polished. Samples for SEM observation were coated with carbon or gold to increase electron conductivity. Observations were performed using a Zeiss Ultra 55 SEM equipped with a field emission gun and a Hitachi S-300 N. Images were acquired with the microscopes operating at 15 kV and a working distance of ~7.5 mm and 15 mm, respectively. Several anuran samples (8 months) were also observed using an environmental SEM FEI QUANTA 200. In this case, fixation was not necessary. The size of cells observed by SEM was analysed afterwards with the imaging software Cell-A 3.0. In order to monitor the conservation state of inner tissues, T2-weighted MRI, performed with a Bruker BMT 47/40 MRI scanner, allowed the observation of several fish bodies prior to fixation and resin embedding. MRI is a non-destructive technique that provides good contrast between the body parts and tissues.

## Additional Information

**How to cite this article**: Iniesto, M. *et al.* Involvement of microbial mats in early fossilization by decay delay and formation of impressions and replicas of vertebrates and invertebrates. *Sci. Rep.* **6**, 25716; doi: 10.1038/srep25716 (2016).

## Supplementary Material

Supplementary Information
