# Supplementary material for: Involvement of microbial mats in early fossilization by decay delay and formation of impressions and replicas of vertebrates and invertebrates
Source: Sci Rep. 2016 May 10;6:25716. doi: 10.1038/srep25716 (PMC4861970; doi:10.1038/srep25716)
Supplement: Supplementary Information [file srep25716-s1.pdf]

Involvement of microbial mats in early fossilization by decay delay and formation of impressions and replicas of vertebrates and invertebrates

Miguel Iniesto[a*], Ángela D. Buscalioni[b], M[a] Carmen Guerrero[a], Karim Benzerara[c], David Moreira[d] and Ana I. López-Archilla[a]

[a]Department of Ecology, Universidad Autónoma de Madrid, 28049, Madrid, Spain; [b]Department of Biology, Universidad Autónoma de Madrid, 28049, Madrid Spain; [c]Institut de Minéralogie, de Physique des Matériaux, et de Cosmochimie, Sorbonne Universités, Centre National de la Recherche Scientifique, Unité Mixte de Recherche 7590, Université Pierre et Marie Curie Paris 06, Muséum National d'Histoire Naturelle, Institut de Recherche pour le Développement Unité Mixte de Recherche 206, 75005 Paris, France; and [d]Unité d'Ecologie, Systématique et Evolution, Centre National de la Recherche Scientifique, Unité Mixte de Recherche 8079, Université Paris-Sud, Université Paris-Saclay, 91405 Orsay Cedex, France

Keywords: exceptional preservation; experimental taphonomy; microbial sarcophagus; microbial entombment; moulds; soft-tissue persistence

*Correspondence to miguel.iniesto@uam.es

Supplementary Fig. S1: The diagram summarizes the sampling and several relevant events monitored during decay of flies (A), fish (B) and frogs (C) in mats (green) and controls over sediment (orange). Each arrow represents at least two bodies analysed in order to describe the decay state.

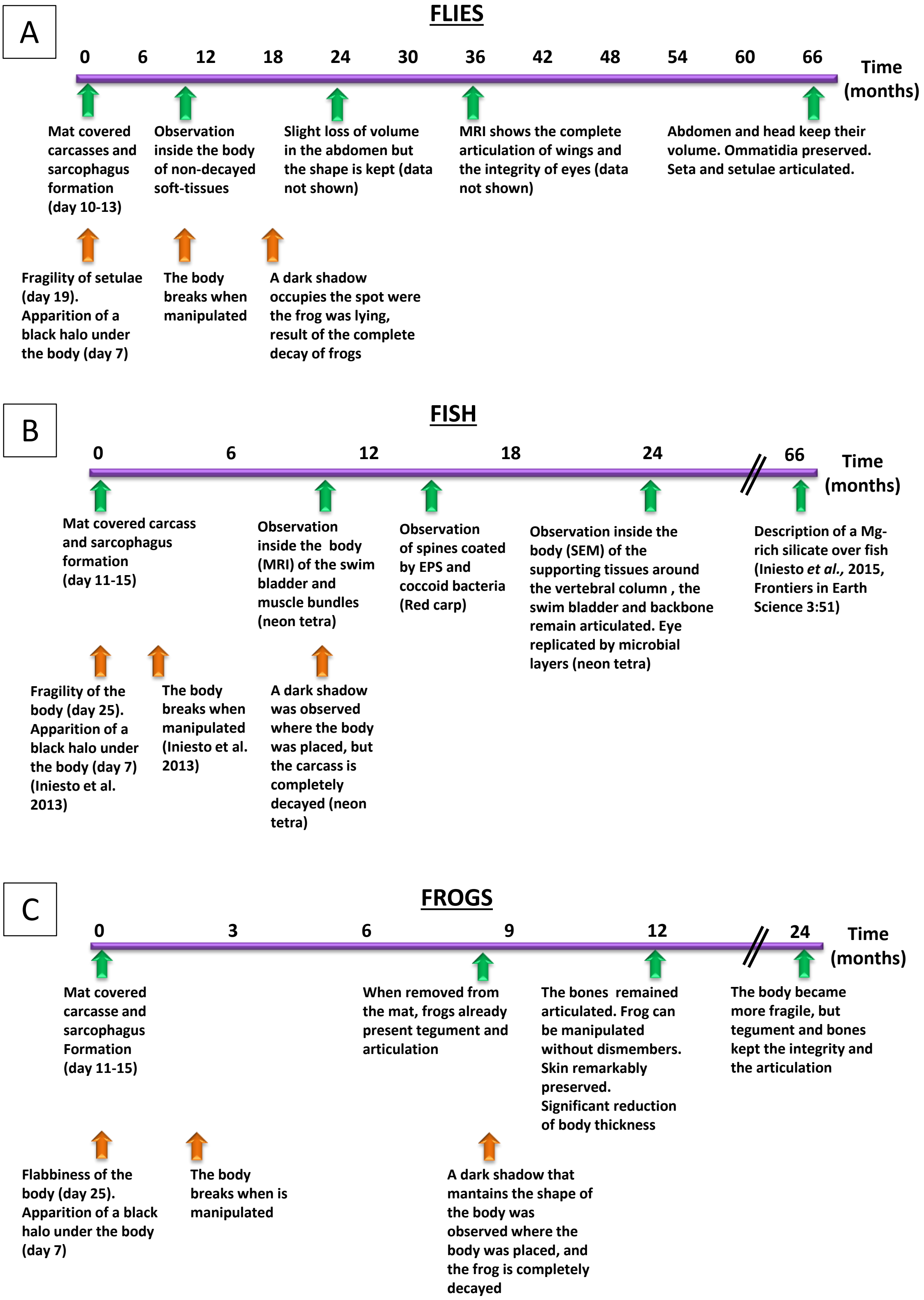

Supplementary Fig. S2: Energy-dispersive X-ray spectrometry (EDXS) of scales of neon tetra fish and the impression generated in the microbial mat.

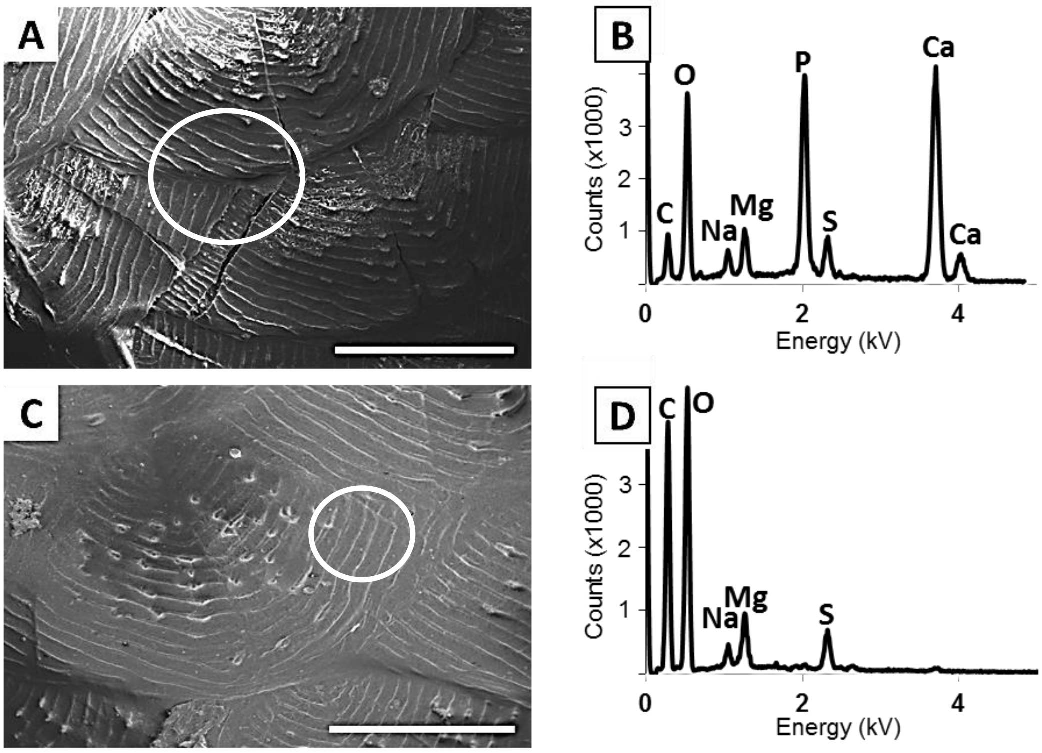

(A) Scales of red carp and the corresponding EDXS spectrum (B). (C) Impression generated in the microbial mat and its EDXS spectrum (D). EDXS confirmed the mineral composition of the scales, made mainly of calcium phosphate, in contrast with the organic composition of this impression. Scale bars: 500 µm.

Supplementary Fig. S3: Integrity of frog carcasses while covered by mats after 12 months

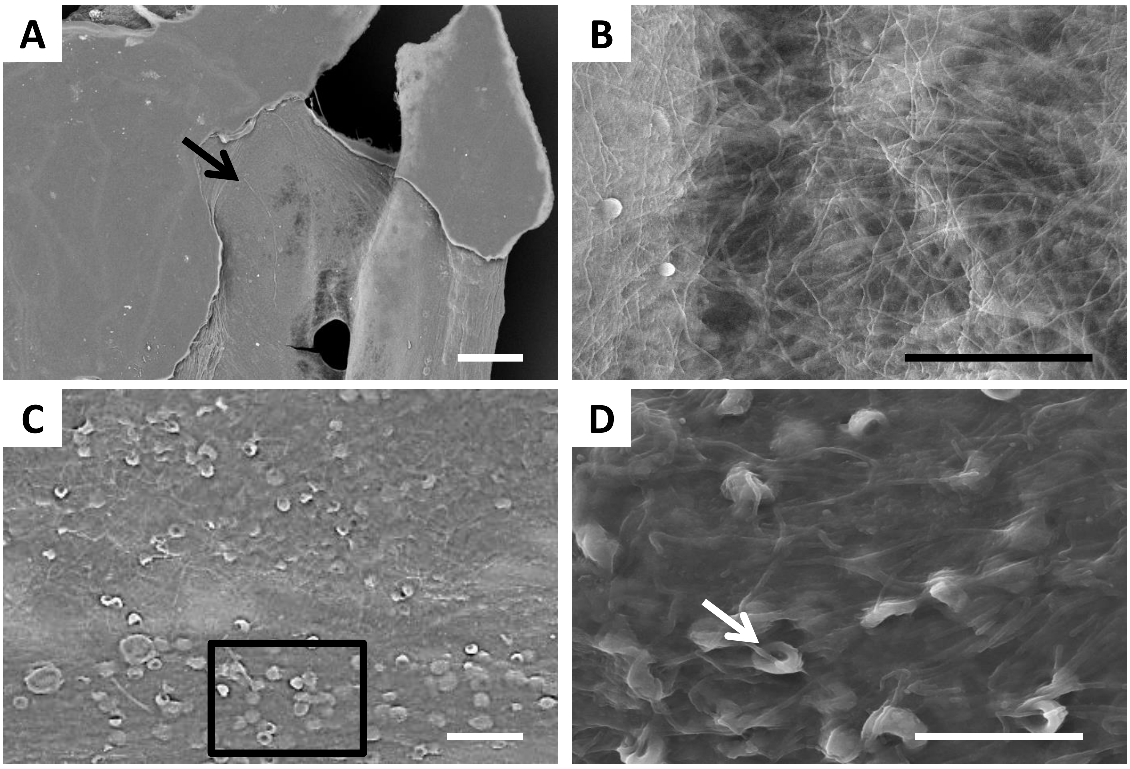

(A) Interdigital webbing of the frog. The surface is partially covered by the mat, but the integrity is remarkable in those zones in which the membrane is exposed (arrow). (B) Skin of the frog at higher magnification, where skin-deep blood vessels are evident. (C) Surface of the leg of frog with skin still covering the appendix. (D) Detail of the surface showed in C. A large number of microbial cells are embedded in a matrix that covers the tegument, and small protuberances of the skin (arrow) rise to the surface despite the microbial film after 12 months. (Scale bar: A, 200 µm; B, 50 µm; C, 70 µm; D, 30 µm)

Supplementary Fig. S4: Schematic diagram of the preservation of carcasses (a fish in the example) into a microbial mat.

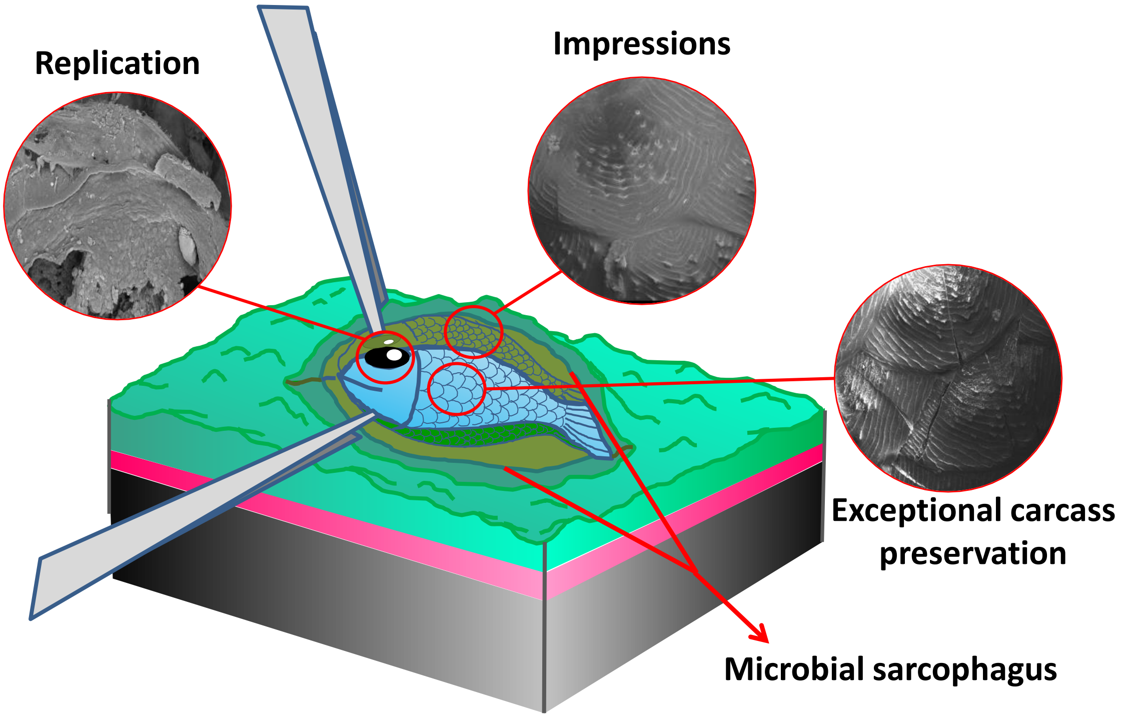

After the coverage of the mat, the intimate contact of the upper layers of the mat and the surface of the body results in the formation of fine impressions and replicas. In addition, the sarcophagus leads to a delay of decay and the preservation of structures such as scales, and even inner soft-tissues.

Supplementary Table S1: Data of the measuring of the different cells observed at SEM

	N	Meaning Value	SD
**Cells in Fly Mould**	118	0.828	0.133
**Cells over scales**	270	0.802	0.165
**Small Cells over Fish Scales (<0.85)**	159	0.701	0.107
**Medium Cells over Fish Scales (>0.85)**	111	0.959	0.088
**Cells over Fish Bones**	130	0.852	0.134
**Filaments in Frog Mould**	74	2.267	0.206
**Filaments from the upper layers**	65	12.77	2.11
**Cells from the upper layers**	25	3.65	0.79
